# Promoting health literacy and sense of coherence in primary care patients with long-term impaired work ability—a pilot study

**DOI:** 10.1080/02813432.2022.2159191

**Published:** 2023-01-09

**Authors:** Märit Löfgren, Gun Rembeck, Dominique Hange, Cecilia Björkelund, Irene Svenningsson, Lena Nordeman

**Affiliations:** a Primary Health Care, School of Public Health and Community Medicine, Institute of Medicine, Sahlgrenska Academy, University of Gothenburg, Gothenburg, Sweden; b Region Västra Götaland, Research, Education, Development & Innovation Primary Health Care, Research, Education, Development & Innovation Center Södra Älvsborg, Borås, Sweden; c Region Västra Götaland, Regional Health, Borås Youth Guidance Center, Borås, Sweden; d Region Västra Götaland, Research, Education, Development & Innovation Primary Health Care, Research, Education, Development & Innovation Center Skaraborg, Skövde, Sweden; e Region Västra Götaland, Research, Education, Development & Innovation Primary Health Care, Research, Education, Development & Innovation Center Fyrbodal, Vänersborg, Sweden; f Unit of Physiotherapy, Department of Health and Rehabilitation, Institute of Neuroscience and Physiology, Sahlgrenska Academy, University of Gothenburg, Gothenburg, Sweden

**Keywords:** Primary health care, sick leave, sense of coherence, health literacy, quality of life

## Abstract

**Objective:**

Evaluate feasibility, partnerships, and study design of intervention to minimise sick leave.

**Design and setting:**

The design was a pilot single arm intervention study in primary health care. Outcome measures at follow-ups for each participant were compared with baseline data for the same person.

**Subjects:**

Twenty primary health care patients with recurrent or long-term sick leave or health-related unemployment.

**Intervention:**

Patient education through interactive study groups that met half a day a week for eight subsequent weeks. Groups were led by experienced but not medically trained facilitators. The intervention was designed to improve participant health literacy, sense of coherence, health-related quality of life, and patient involvement in healthcare.

**Main outcome measures:**

Primary outcome was the level of sick leave. Sick leave data were obtained from medical records when available, otherwise patient reported. Secondary outcomes regarding health literacy, sense of coherence, and health-related quality of life were measured with validated questionnaires at baseline and follow-ups.

**Results:**

Level of sick leave decreased significantly and participation in work preparatory activities increased during follow-up. Health literacy, sense of coherence (subscale sense of meaningfulness), and health-related quality of life (subscale social functioning) showed statistically significant improvement. Intervention, partnerships, and study design were feasible.

**Conclusion:**

An educational programme, conducted in cooperation between primary health care and partners outside the healthcare system, was feasible and showed an impact on sick leave, health literacy, sense of coherence, and health-related quality of life.

**KEY FINDINGS**
- A pilot study to evaluate an educational programme with study groups conducted in cooperation between primary health care and partners outside the healthcare system showed good feasibility.
- Sick leave decreased significantly six months after baseline.
- Health literacy, sense of coherence (subscale sense of meaningfulness), and health-related quality of life (subscale social function) improved significantly 6 months after baseline.

## Background

Long-term health-related inability to work is a major problem in Sweden despite reduction efforts, and return-to-work rates from long-term illness are very low [1]. Findings from previous research are complex, as fewer symptoms and increased well-being are not necessarily associated with reduced sickness absence [2], non-medical factors may influence sickness certification [3], and educating physicians in sickness certification practice has not shown robust effects on return-to-work rates [4].

This study evaluates a Swedish employee-driven initiative to improve sick leave and the rehabilitation process among frequent attenders with health-related impaired work ability at primary health care. Preparatory interviews with sick leave and rehabilitation process stakeholders, including the Social Security Office, indicated that these patients experienced repeated process related disappointments and loss of control leading to inactive coping strategies and poor health outcomes. Improved interactions between patients and key actors to find solutions to overcome health-related impairment might conceivably be a way to influence process outcomes. A literature search was conducted to identify a theoretical framework for an intervention. The search was not done systematically as is ideal [5], but the value of stakeholder engagement in developing complex interventions is highlighted in updated guidelines [6].

Previous research shows that illness perception is a complex phenomenon with biological, psychological, and social dimensions [7]. The same medical diagnosis may have different consequences for different individuals [8]. Psychiatric comorbidity, which is common among patients with chronic illness, has a negative effect on health outcome and return to work [8–10]. Moreover, health is not just the absence of disease, but also the ability to adjust to change [11].

Sense of coherence, i.e. finding life comprehensible, manageable and meaningful, protects against malaise due to external stress, such as disease [12]. Low sense of coherence is associated with several factors defining the target population, such as frequent attendance at health care [13], poor adaptation to illness, and increased comorbidity with depression [14]. Sense of coherence is important for working life [15], and low sense of coherence may predict long-term sick leave [16–21].

Patient involvement in healthcare decisions improves adherence, patient satisfaction, and health outcomes [22,23]. Health literacy includes abilities needed to make good decisions about health care and is affected by cognitive status, educational attainment, communicative skills, socioeconomic situation, and context [24,25].

Sick leave entails loss of control and has a negative impact on the person’s social situation, whereas working promotes health through financial security, improved self-esteem, and social relationships [26–29]. Social recovery is crucial for leading a meaningful life despite experiencing ill health [30]. Peer support increases social functioning and has a positive effect on the ability to work despite long-term illness [26,31].

Knowledge about which interventions have an impact on sick leave is insufficient, but there is some evidence supporting interventions in close collaboration with the workplace early in the sick leave and rehabilitation process [29,32]. Effectiveness of para-medical interventions focusing on enhancing patient resilience and coping on return-to-work is uncertain [33,34], and there is much yet to be understood about social recovery [30]. Both health literacy and sense of coherence may be affected by interventions [35,36], but no randomized controlled trial focusing on increasing health literacy or sense of coherence to reduce sick leave was found.

Assuming that most patients want to work if they perceive they can, this pilot study evaluates a patient educational programme designed to increase health literacy and sense of coherence to improve patient participation in decisions related to sick leave and rehabilitation in primary health care. Focus was on helping patients describe their individual needs and barriers to working (and living a meaningful life) and to enhance their problem-solving communication with sick leave and rehabilitation process actors so that each participant could obtain person-centred support for realising goals in life and at work. Unlike previously studied psycho-educational or multidisciplinary rehabilitation interventions, the present intervention was largely non-medical, focusing on communication skills and practical problem-solving abilities in the individual context of the sick leave and rehabilitation process.

The present pilot study was performed to evaluate the intervention and study design and collect information for designing a randomized controlled trial.

## Purpose

Feasibility testing of a patient educational programme to evaluate the intervention, partnerships, and study design to obtain information regarding whether to proceed with a full-scale randomized controlled trial to investigate the intervention’s effect on sick leave, health literacy, sense of coherence and health-related quality of life (HRQoL).

## Materials and methods

### Study design and selection of participants

The design was a pilot single arm intervention study. Outcome measures at follow-up for each participant were compared with baseline data for the same person. Primary outcome was level of sick leave. Data about sick leave and health-related unemployment before baseline were patient-reported when medical records were incomplete. Follow-up data were obtained from medical records after three and six months. Secondary outcomes were health literacy, sense of coherence, and HRQoL, measured by validated questionnaires.

The target group consisted of primary health care patients with persisting impaired work ability resulting in long-term or recurrent sick leave or health-related unemployment. Patients were asked to participate in the patient educational programme based on repeated attendance for similar health problems impairing work ability in combination with difficulties in finding medical or vocational rehabilitation suitable to patient needs due to recurrent symptoms or passive coping strategies. Eligibility for inclusion in the study was not dependent on any specific diagnosis. There was no upper limit on duration of sick leave.

Physicians in primary health care referred patients to the educational programme. It was optional for programme patients to participate in the research study. All eligible patients present at the first group meeting for each study group were informed of the research study and asked for consent to participate. Written informed consent was obtained from all research subjects. The Regional Ethical Review Board in Gothenburg approved the study (DNR 645-15).

### Intervention

Four study groups with 7–10 consecutively recruited participants (among which 20 research subjects) met half a day a week for eight subsequent weeks. No consideration was taken regarding group composition. Group leadership was shared by two experienced, but not medically trained, supervisors. GPs were informed about the intervention aim and encouraged to ask patients about their action plans.

The patient educational programme strategy was to increase:

1. *Health literacy* by supervisor-led sessions about:
  - Bodily reactions to stress and long-term pain. Long-term disease as a stress factor.
  - The impact of healthy lifestyles on HRQoL. Strategies for increasing well-being despite illness.
  - Finding evidence-based information and support to make good health-related decisions.
  - Roles and responsibilities of different sick leave and rehabilitation process stakeholders and available vocational rehabilitation.
  - Tools and strategies for solving problems, managing change, and reaching goals. Working with a personal action plan.
  - Communication strategies to enable good relations with authorities, employers, family, and friends to solve health-related problems.
2. *Sense of coherence* by facilitating a dialogue between peers and individual reflection about:
  - Factors influencing individual well-being (comprehensibility of symptoms from a bio-psycho-social perspective).
  - Factors influencing individual ways of communicating and how to improve these (comprehensibility of the origin of conflicts and benevolent treatment from officials and family, manageability of personal relations despite illness).
  - Adapting personal drivers and preferences in life to present life circumstances (finding meaningfulness in life despite illness).
  - Enabling conscious decisions about health, rehabilitation, and return-to-work based on own preferences in private and working life (manageability and control).

The patient educational programme was available at one primary health care centre in Borås, Sweden, from fall 2015 to spring 2016.

### Theoretical mechanisms of action

The intended intervention is a complex intervention (3). There are several intervention elements that may contribute to decreased sick leave: (i) the educational content; (ii) the way the content was delivered *via* study groups; (iii) increased health literacy and/or sense of coherence; and iv) available support from primary health care, case manager and employer in solving problems related to rehabilitation and return-to-work in the individual context.

### Cooperation with actors outside the healthcare system

In Sweden, there are several educational associations receiving state and municipal funding for educational and cultural activities. The pedagogic method of these educational associations is the study group, i.e. interactive learning in small supervised groups following a study plan.

The patient educational programme was conducted in the form of supervised study groups in collaboration between primary health care and an educational association. Primary health care contributed with evidence-based knowledge, chose the study group supervisors based on previous experience, formal education, and personal commitment, and provided them with training.

The study group supervisors did not have medical training but previous experience from group coaching, teaching communication skills in business environments, and community initiatives aimed at the target group. Before starting, they received training in pedagogic methods, educational programme content, and background knowledge to be able to use the material in group coaching.

### Data collection

Background data concerning age, sex, education, occupation, and previous long-term sick leave (occurrence of previous sick leave >3 months yes/no) were collected at baseline. Data for all research variables were collected at baseline and followed up three and six months after baseline.

The primary outcome measure was sick leave level, defined in Sweden as the percentage of regular working hours the person is currently on sick leave, i.e. 25, 50, 75, or 100%. The measure was chosen to enable assessing intra-individual changes in sick leave from baseline to follow-up as the research subjects were their own controls. Data on history of sick leave and health-related unemployment before baseline were patient-reported when medical records were incomplete regarding this information. Follow-up data were obtained from medical records. Participation in work-related activities (such as work preparatory rehabilitation programmes) while on sick leave benefit was registered at all measurement points.

**Figure 1.**
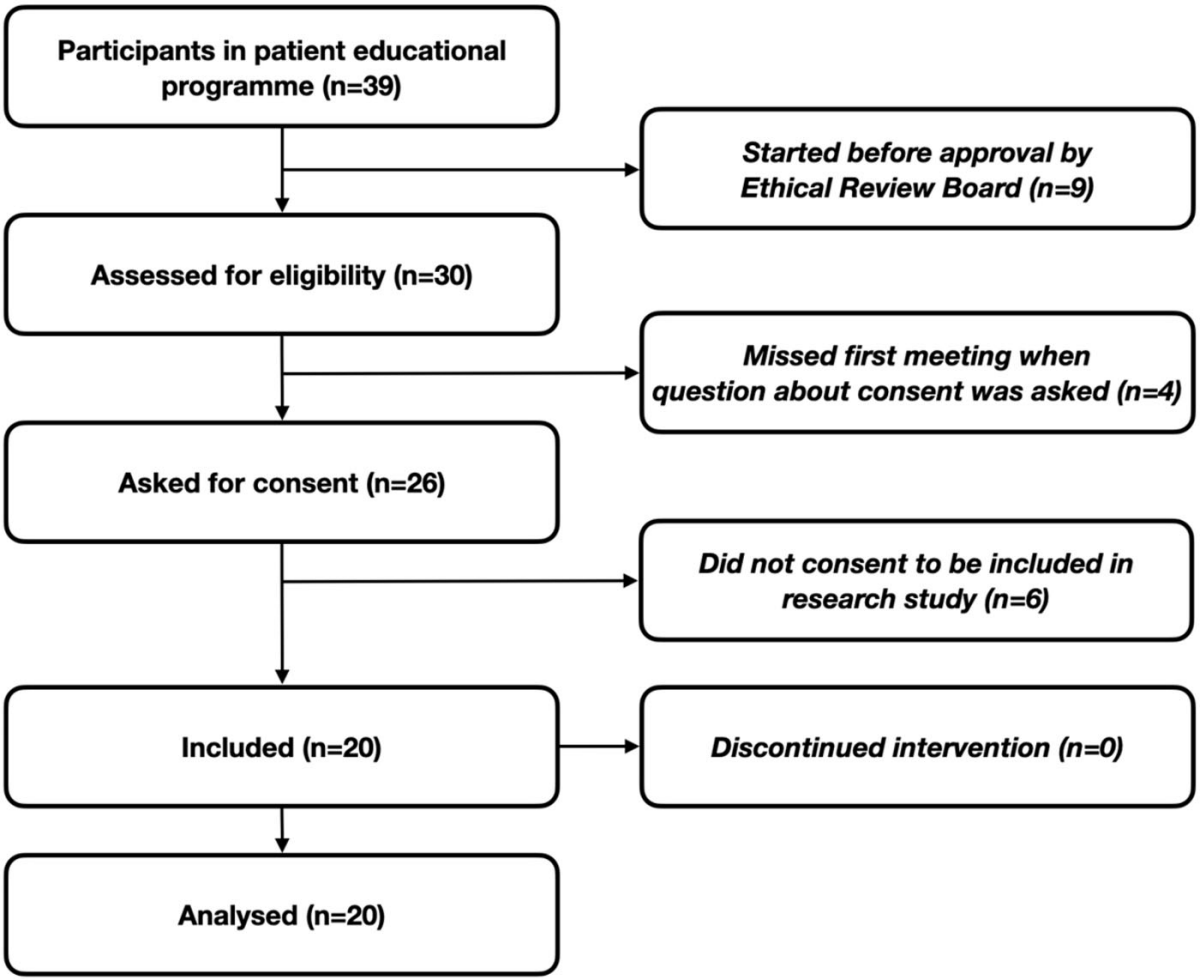
Participants in patient education programme.

Validated questionnaires were used to measure secondary outcomes, health literacy, sense of coherence, and HRQoL. Health literacy was measured with HLS-EU-Q16 Swedish version [37]. Sense of coherence was measured with SOC-29 [12], and HRQoL with SF-36/RAND-36 [38]. The recruitment process is documented in Figure 1.

Participants’ reception of the intervention was analysed based on interest in participating in the intervention, number of dropouts, and presence at study circle meetings.

### Statistical analysis

Outcome data were analysed and presented descriptively. Answers to questionnaires were compiled according to a manual for each questionnaire. Health literacy was defined as insufficient (0–8p), problematic (9–12p), or sufficient (13–16p) [37]. The participants’ average sense of coherence value was compared to a Swedish urban population [39]. HRQoL values were compared to a mixed Swedish population aged 30–59 years [40].

For each research subject, baseline data were compared with data after 3 and 6 months, respectively, to register the direction of change between the follow-up points. Wilcoxon Signed Ranks Test was used to analyse intra-individual direction of change between baseline and follow-ups on group level. The effect size was calculated using Cohen’s *d. p*-Value was set to <0.05. Statistical analyses were performed using the statistics program SPSS (IBM, SPSS for Windows version 21.0).

## Results

### Feasibility of partnerships and pedagogics

Collaborations between primary health care, the educational association, and the group supervisors proceeded without friction. Study group supervisors felt comfortable following the study plan stipulated by primary health care, making good use of their previous experience from group coaching and teaching communication skills. The interactive pedagogic method of the study group allowed the adjustment of meeting time allocation according to each patient group’s preferences. Individual medical questions were referred to primary health care.

### Selection, recruitment, and data availability

Four consecutive patient study groups were conducted during a 10-month period with patients from one primary care centre with 14,000 listed patients. In total, 44 persons were registered to participate in the patient educational programme, among which 39 persons (89%) started and 34 persons (77%) completed the programme. On average, patients attended 7 of 8 (>85%) study group meetings (dropouts excluded).

Thirty patients were assessed for eligibility to participate in the present research study, among which 26 were asked for consent to participate, and 20 patients (77%) were included in the study (Figure 1).

Background information about the level of education was missing for five participants (25%). Information about the level of sick leave, employment, and participation in work preparatory activities was available for all participants at all measurement points. Secondary outcome data were complete for 19 out of 20 participants at baseline, and data from two measurement points were available for 15 (75%) research subjects.

### Participants at baseline

Participants were 23-63 years old and 70% were females. Half had more than 12 years of education and nearly all (95%) had a history of previous long-term sick leave. The majority (65%) had suffered from health-related impaired work ability for more than 12 consecutive months. Diagnoses mostly included recurrent depression and/or anxiety disorders and/or musculoskeletal pain. A majority had permanent jobs, 20% had sheltered employment, and 20% were unemployed (Table 1).

**Table 1.** Participants in the pilot study (*n* = 20) at baseline.

	Women	Men	Total
Age, mean (*SD*)	47.9 (11.7)	41.5 (14.4)	46.0 (12.6)
	% (*n*/*n*)	% (*n*/*n*)	% (*n*/*n*)
Sex	70% (14/20)	30% (6/20)	100% (20/20)
Previous long-term sick leave	100% (14/14)	83% (5/6)	95% (19/20)
Highest level of education			
Incomplete elementary school	0% (0/14)	33% (2/6)	13% (2/15)
Elementary school	7% (1/14)	17% (1/6)	13% (2/15)
Senior high school	21% (3/14)	0% (0/6)	20% (3/15)
University	50% (7/14)	17% /1/6)	53% (8/15)
Employment			
Full time work	64% (9/14)	33% (2/6)	55% (11/20)
Part time work	7% (1/14)	0% (0/6)	5% (1/20)
Sheltered employment	14% (2/14)	33% (2/6)	20% (4/20)
Unemployed	14% (2/14)	33% (2/6)	20% (4/20)

Seventy percent were on full-time or part-time sick leave. Twenty percent were unemployed and had repeatedly sought health care for long-term health-related work impairment. The remainder had either a history of previous long-term sick leave and had been referred to participate in the intervention as an alternative to sick leave or had self-selected part-time work due to ill health (Table 2).

**Table 2.** Change in employment status and level of sick leave for the participants in the pilot study (*n* = 20) from baseline to 3 and 6 months.

Employment status	Baseline % (*n*/*n*)	3 months % (*n*/*n*)	6 months % (*n*/*n*)
Full-time sick leave	40% (8/20)^a^	35% (7/20)	25% (5/20)
Part-time sick leave	30% (6/20)^b^	35% (7/20)	35% (7/20)
Currently working	5% (1/20)	5% (1/20)	15% (3/20)
Self-selected part-time^c^	5% (1/20)	5% (1/20)	5% (1/20)
Enrolled at the employment office^d^	10% (2/20)	15% (3/20)	20% (4/20)
Provided for by relatives^e^	10% (2/20)	5% (1/20)	0% (0/20)

Outcome variable	Baseline^f^	3 months^f^	*p*-Value	6 months^f^	*p*-Value
Level of sick leave (*n* = 20)^g,h^	55 (43); 50 (0–100)	54 (42); 50 (0–100)	0.79	39 (41); 25 (0–88)	**0.016** ^i^
Level of sick leave (*n* = 14)^g,j^	79 (26); 100 (50–100)	77 (25); 88 (50–100)	0.79	55 (38); 50 (25–100)	**0.016** ^i^

^a^Duration of sick leave more than 12 months *n* = 5, 6–12 months *n* = 1, 3–6 months *n* = 2, full-time employed *n* = 6, sheltered employment *n* = 1, unemployed *n* = 1.

^b^Duration of sick leave more than 12 months *n* = 3, 6–12 months *n* = 1, 3–6 months *n* = 2, full-time employed *n* = 4, sheltered employment *n* = 2.

^c^Due to ill health.

^d^Participants with sheltered employments not included (*n* = 4).

^e^Unemployed, not looking for a job or participating in work preparatory activities due to health related work impairment since more than 12 months.

^f^Mean (*SD*); median (25:e–75:e percentile).

^g^Level of sick leave means percentage of regular working hours the person is currently on sick leave (25, 50, 75, or 100%).

^h^Participants who are not on sick leave are counted as 0% level of sick leave (*n* = 6).

^i^Bold text indicates significant results, i.e. *p*-value <0.05.

^j^Participants who are not on sick leave not included (*n* = 6).

Health literacy was insufficient or problematic for half the group. The average sense of coherence value was more than two *SD* below the reference population. The participants scored much lower than the reference population on all subscales of HRQoL (Table 3).

**Table 3.** Change in health literacy, sense of coherence, and health-related quality of life for the participants in the pilot study (*n* = 20) from baseline to 3 and 6 months follow-up, respectively.

Health literacy^a^	Baseline (*n* = 19)	3 months (*n* = 12)	*p*-Value	6 months (*n* = 10)	*p*-Value
Total	12 (4); 12 (8–15)^b^	14 (4); 16 (12–16)^b^	0.63	14 (2); 15 (14–16)^b^	**0.029** ^f^
Sufficient health literacy	47% (9/19)^c^	67% (8/12)^c^		90% (9/10)^c^	
Problematic health literacy	26% (5/19)^c^	17% (2/12)^c^		10% (1/10)^c^	
Insufficient health literacy	26% (5/19)^c^	17% (2/12)^c^		0% (0/10)^c^	
Sense of coherence^d^	Baseline (*n* = 20)^b^	3 months (*n* = 12)^b^	*p*-Value	6 months (*n* = 9)^b^	*p*-Value
Total	108 (27); 107 (92–121)	116 (27); 119 (101–137)	0.21	112 (25); 106 (93–129)	0.15
Comprehensibility	39 (9); 39 (32–44)	41 (10); 44 (34–50)	0.43	40 (12); 45 (29–47)	0.44
Manageability	38 (10); 40 (32–44)	41 (9); 41 (34–49)	0.21	38 (9); 38 (31–43)	1.00
Meaningfulness	31 (11); 30 (23–36)	34 (10); 35 (26–41)	0.13	34 (8); 35 (29–39)	**0.024** ^f^
Health-related quality of life^e^	Baseline (*n* = 19)^b^	3 months (*n* = 12)^b^	*p*-Value	6 months (*n* = 11)^b^	*p*-Value
Physical functioning	68 (23); 70 (60–85)	73 (17); 71 (66–84)	0.91	73 (18); 75 (55–85)	0.36
Role functioning/physical	28 (36); 25 (0–50)	10 (23); 0 (0–19)	0.084	30 (46); 0 (1–100)	0.58
Bodily pain	47 (28); 45 (23–68)	54 (30; 56 (23–70)	1.00	49 (23); 45 (29–68)	0.50
General health	39 (20); 40 (25–50)	48 (22); 50 (31–68)	0.53	39 (21); 35 (25–55)	0.92
Energy/fatigue	28 (17); 30 (15–35)	33 (20); 49 (30–64)	0.04	31 (22); 27 (15–45)	0.47
Social functioning	41 (17); 38 (25–50)	48 (27); 50 (28–59)	0.17	59 (27); 50 (38–75)	**0.023** ^f^
Role functioning/emotional	12 (20); 0 (0–33)	19 (39); 0 (0–17)	0.58	30 (38); 33 (0–33)	0.53
Mental health	44 (22); 48 (24–60)	51 (18); 49 (37–68)	0.12	44 (23); 40 (24–64)	0.95
Health change	36 (30); 25 (0–75)	40 (38); 50 (0–75)	0.74	45 (37); 50 (0–75)	0.16

^a^HLS-EU-Q10 Swedish version. Range 0–16p. Higher number indicates better health literacy.

^b^Mean (*SD*); median (25:e–75:e percentile).

^c^% (*n*/*n*).

^d^SOC-29, total score 203p, three subscales, comprehensibility 11–77p, manageability 10–70p, meaningfulness 8–56p, higher number indicates higher SOC.

^e^RAND 36/SF 36 with nine subscales, range 0–100p each subscale, higher number indicates better health-related quality of life.

^f^Bold text indicates significant results, i.e. *p*-value <0.05.

### Three months follow-up

There was no difference in the level of sick leave after 3 months. One unemployed participant had enrolled at the employment office looking for a full-time job (Table 2).

Health literacy, sense of coherence, HRQoL, and attitudes had generally improved, except for HRQoL role functioning/physical, but the differences were not statistically significant (Table 3).

### Six months follow-up

Looking only at patients on sick leave (*n* = 14), there was a 24% decrease in the mean level of sick leave after 6 months (Table 2). Using Cohen’s calculation to relate the size of the change in the level of sick leave to the standard deviation (*SD*), the effect size ranged between 0.63 (medium) and 0.92 (large) depending on whether using *SD*_6 months_ or *SD*_baseline_. No participant had increased their level of sick leave compared to baseline. Additionally, two persons still on full-time sick leave were participating in work preparatory activities compared to baseline. The unemployed participant enrolled at the employment office was working full-time, and the person who was referred to the intervention as an alternative to sick leave never needed sick leave (Table 2).

There was a statistically significant positive change in health literacy, sense of coherence subscale sense of meaningfulness, and HRQoL subscale social function compared to baseline. The other subscales showed non-significant positive change or no change (Table 3). No conclusions about possible correlations between health literacy, sense of coherence, HRQoL, and level of sick leave could be drawn.

## Discussion

This study evaluates feasibility, partnerships, and study design of a new educational programme with the aim to minimise sick leave based on collaboration between primary health care and partners outside the healthcare system. Collaborations worked well. Participant attendance at group meetings was high and the participants were active during the meetings. As participation was voluntary and unpaid, this indicates that there was an interest in the intervention. It was easy to recruit subjects to the research study, but despite no dropouts from the intervention, there were subjects lost to follow-up.

The level of sick leave decreased significantly, and participation in work preparatory activities increased during the study period. Secondary outcomes health literacy, sense of coherence, and HRQoL showed an overall trend towards improvement. Results for health literacy and the subscales sense of meaningfulness (sense of coherence) and social functioning (HRQoL) showed statistically significant improvement.

### Strengths and weaknesses

The study design was chosen to assess the feasibility of the intervention, partnerships, and intervention design. In the absence of a control group, the study could not reach any conclusions on the effectiveness of the intervention. However, both primary and secondary outcomes changed in positive directions, albeit this needs to be evaluated further in a randomized controlled trial.

A weakness of the study is that the collaborating partners’ and patients’ experiences of the intervention were not evaluated systematically. A strength of the study is that referral of participants to the intervention was based on need rather than on specific diagnosis, as this may enhance the results’ relevance for a primary care setting.

### Interpretation of results and comparison with other studies

Participants’ health literacy and sense of coherence at baseline were below average, as anticipated. A majority of the participants were women, reflecting the gender distribution of patients in primary health care [41]. Both the percentage of well-educated patients and the percentage with low education were higher than for the Swedish reference population [42], and the percentage of patients with sheltered or no employment was high [43].

Participants were actively listening, discussing, and offering each other peer support during group sessions, and professionals reported that patients were more active in planning rehabilitation and work preparatory activities post-intervention. The level of sick leave decreased during follow-up, as was hypothesised, but not significantly until after six months, which could be due to process-related delays in the rehabilitation process. Health literacy and sense of meaningfulness (sub scale of sense of coherence) increased, which is in concordance with previous findings that health literacy and sense of coherence may be influenced by interventions [35,36]. However, changes could be a result of the intervention or an effect of treatment as usual in primary health care, case management as usual by the Social Insurance Agency, a natural course of events, or as regression towards the mean. A randomized controlled trial is needed to study intervention effects.

### Considerations for a future randomized controlled trial

Participants’ health literacy, sense of coherence, and level of sick leave changed in the expected direction, and the patient educational programme was well received by participants. Hence, no major programme revision is needed.

The evaluation method with questionnaires seems to have captured the outcomes since there was a significant positive change in study variables despite few participating patients. This supports the use of this measurement method in a future randomized controlled trial. As there were subjects lost to follow-up, a research coordinator will be needed to increase the response rate by reminding participants to complete the follow-up assessments. The change in outcomes observed in this pilot study means that sample sizes to detect clinically relevant change with pre-defined statistical power may be calculated for the main study.

In the pilot study, the primary outcome measure, level of sick leave, enabled research subjects to serve as their own controls. In a future randomized controlled trial, it is recommended to measure net days with sick leave to compare the intervention arm with control arm, and sick leave data should be retrieved from the Social Insurance Agency.

Data did not allow any conclusions to be drawn about correlations between health literacy, sense of coherence, HRQoL, and level of sick leave. Not all participants were on sick leave at baseline as some were unemployed due to impaired health. Most participants in the pilot study (80%) updated their rehabilitation plans to include vocational rehabilitation, for example, work training, without changing their work status. Such change in activity level will not be captured by measuring sick leave. Therefore, in a randomized controlled trial, it is recommended to register participation in vocational rehabilitation, in addition to sick leave, to capture increase in activity level despite no change in the level of sick leave. To better understand correlations between primary and secondary outcomes, a mediation analysis is recommended.

Sick leave was chosen as the primary outcome to relate the results to a possible societal benefit to accommodate decision makers’ agenda. However, the target group is known to suffer, and the intervention might be worthwhile even if sick leave is not reduced. Thus, we suggest adding symptoms of depression/anxiety/exhaustion/chronic pain as secondary outcomes in a randomized controlled trial.

Inclusion criteria were chosen to select patients in the sick leave and rehabilitation process who did not find their health conditions comprehensible or manageable (indicating low health literacy and low sense of coherence), who were currently not involved in decisions about rehabilitation and return-to-work and who were lacking a realistic person-centered rehabilitation plan. The patient group studied was heterogeneous with respect to medical history, baseline data, and scoring on health literacy and sense of coherence tests. Since the inclusion criteria were selected based on stakeholder interviews, and it was easy to find study patients, the criteria are perceived to be relevant for sick leave and rehabilitation process patients in a primary health care setting. Narrowing the inclusion criteria would allow for a more homogeneous target group, but the results would presumably be less applicable in a primary health care context. For a future randomized controlled trial, it is recommended to define the target group by a lower inactivity level due to sick leave or health-related unemployment, e.g. 60 net days during the preceding 6 months, yet to keep the broad inclusion criteria for research relevance in a primary health care setting.

The cost of sick leave in Sweden was 6.3 billion € in 2020 [44]. For diagnoses common in the study population, i.e. recurrent depression and/or anxiety disorders and/or chronic non-cancerous pain, sick leave for more than one year is within the range of what is accepted in Sweden [45]. The cost per patient in the intervention was 7–800 € (depending on group size), a cost comparable to the cost of one week of sick leave based on the mean salary in Sweden [46]. To evaluate the cost-effectiveness of the intervention, it would be important to perform a cost-effectiveness study in parallel with a randomized controlled trial.

## Conclusions and implications

Based on findings from this pilot study showing decreased work absence and significant improvement in health literacy, sense of coherence subscale meaningfulness, and HRQoL subscale social functioning, the recommendation is to further evaluate the intervention in a randomized controlled trial.

The intervention was conducted in collaboration between primary health care and partners outside the healthcare system and the study methods used for patient evaluation were feasible with minor changes.

Baseline data for the participants showed low health literacy and low sense of coherence, suggesting that it is important to target health literacy and sense of coherence with interventions for patients with recurrent or long-term sick leave or health-related unemployment. Further research to understand the association between health literacy, sense of coherence, and sick leave is recommended.
